# Weight changes and the incidence of depressive symptom in the middle-aged and older adults: findings from the Chinese nationwide cohort study

**DOI:** 10.1186/s12889-022-14624-5

**Published:** 2022-12-06

**Authors:** Lin Zhang, Jin-long Li, Lei-lei Guo, Guang Xu, Liu Yang, Congzhi Wang, Ting Yuan, Dongmei Zhang, Jing Li, Yunxiao Lei, Lu Sun, Xiaoping Li, Ying Hua, Hengying Che, Haiyang Liu

**Affiliations:** 1 Department of Internal Medicine Nursing, School of Nursing, Wannan Medical College, 22 Wenchang West Road, Higher Education Park, Wuhu City, An Hui Province People’s Republic of China; 2 Department of Occupational and Environmental Health, Key Laboratory of Occupational Health and Safety for Coal Industry in Hebei Province, School of Public Health, North China University of Science and Technology, Tangshan, Hebei Province People’s Republic of China; 3 Department of Surgical Nursing, School of Nursing, Jinzhou Medical University, No.40, Section 3, Songpo Road, Linghe District, Jinzhou City, Liaoning Province People’s Republic of China; 4 Department of Radiotherapy, Third Affiliated Hospital of Jinzhou Medical University, No. 28, Section 2, Chongqing Road, Linghe District, Jinzhou City, Liaoning Province People’s Republic of China; 5 Obstetrics and Gynecology Nursing, School of Nursing, Wannan Medical College, 22 Wenchang West Road, Higher Education Park, Wuhu City, An Hui Province People’s Republic of China; 6 Department of Pediatric Nursing, School of Nursing, Wannan Medical College, 22 Wenchang West Road, Higher Education Park, Wuhu City, An Hui Province People’s Republic of China; 7 Department of Surgical Nursing, School of Nursing, Wannan Medical College, 22 Wenchang West Road, Higher Education Park, Wuhu City, An Hui Province People’s Republic of China; 8 Department of Emergency and Critical Care Nursing, School of Nursing, Wannan Medical College, 22 Wenchang West Road, Higher Education Park, Wuhu City, An Hui Province People’s Republic of China; 9 Rehabilitation Nursing, School of Nursing, Wanna Medical College, 22 Wenchang West Road, Higher Education Park, Wuhu City, An Hui Province People’s Republic of China; 10 Department of Nursing, Yijishan Hospital, the First Affiliated Hospital of Wannan Medical College, Zheshan West Road, Yijishan District, Wuhu City, Anhui Province People’s Republic of China; 11 Student Health Center, Wannan Medical College, 22 Wenchang West Road, Higher Education Park, Wuhu City, An Hui Province People’s Republic of China

**Keywords:** Cohort study, Depressive symptoms, Incidence, Middle-aged and older adults, Weight change

## Abstract

**Background:**

Previous studies, predominantly in Western individuals, have reported weight gain or weight loss are related to the increased depressive symptoms at all ages, but no study of depressive symptoms has examined its relation to actual (not just self-reported) weight changes in the middle-aged and older adults. Evidence of the relationship in older Asian individuals remains sparse. The study aimed to examine the relationship between weight changes and incidence of depressive symptoms in a nationally representative sample of community-dwelling older Asians.

**Method:**

Data were obtained from the China Health and Retirement Longitudinal Study (CHARLS), which included 17,284 adults aged 45 years. Participants were followed every two years using a face-to-face, computer-aided personal interview (CAPI) and structured questionnaire. We excluded participants with no follow-up data. The numbers of individuals who completed both the baseline and follow-up surveys were 3118 for the short-term (two years from 2011–2013) and the long-term (four years from 2011–2015). Additionally, to associate weight change with subsequent depressive symptoms, we also included 2472 participants without depressive symptom in 2013 and observed the incidence of depressive symptom in 2015 (subsequent term from 2013–2015). Finally, weight changes were classified as loss > -3%, stable-3–3%, gain3-6%, gain6-9%, and gain > 9%. Multivariable-adjusted cox regression in the study were used to assess the hazard ratios (HRs) of each weight change category.

**Results:**

The incidence of depressive symptoms was 20.72% in the 2011–2013, 27.04% in the 2011–2015, and 23.02% in 2013–2015. Weight loss > 3% for all intervals was associated with higher depressive symptoms than stable weight during the 2011–2013 [1.305(1.031,1.651)] among the total populations. When stratified by sex, the results in males and females were different from those in the total population [females:1.389(0.997, 1.935); males:1.263(0.902, 1.767)]. Weight loss > 3% for intervals was associated with higher depressive symptoms than stable weight during the 2013–2015[1.643(1.140, 2.368)] among the males and its effect was also stronger for the total in 2011–2013. Moreover, there was no significant association between weight gain and incident depressive symptom, and no significant interaction effect in terms of the sex*weight changes.

**Conclusion:**

Our findings could inform health promotion interventions to body-weight management aimed at improving the health of the middle-aged and older adults, particularly in the total people with short-term weight loss and males with subsequent term weight loss.

## Background

The depressive symptom is a common psychological disorder condition that represents a serious burden worldwide. Approximately 322 million people worldwide suffer from depressive symptom [1]. Weight change over the course of several years is often a good predictor of depressive symptom in adolescence [2] and adult [3]. It has been shown to predict the incidence of depressive symptom better than a single weight assessment, particularly in old age [4–6]. However, previous studies findings on the association between weight change and depressive symptom have been inconsistent. Moreover, the effect of weight change on depressive symptom can be influenced by several factors such as demographic characteristics, living habits, health status, and baseline body mass index.

A recent meta-analysis [7] conducted with 53 studies including 12 cohort studies and 41 cross-sectional studies showed that underweight and obesity increased the risk of depression, and the relationship between overweight and depression differed by sex. Furthermore, the cohort studies showed a significant increase overall hazard ratios (HRs) of depression in underweight, while those showed no statistically significant relationship with depression in overweight. In subgroup analyses, it was found different results according to sex (men: HR = 0.84, 95%CI 0.72–0.97, women: HR = 1.16, 95%CI 1.01–1.25). In cross-sectional studies, obesity with BMI ≥ 40 kg/m^2^ (OR = 1.18, 95%CI 1.11–1.26) showed a greater pooled odds ratio (OR) than obesity with BMI ≥ 30 kg/m^2^(OR = 1.59, 95%CI 1.12–2.24). Although the meta-analysis included cohort studies and cross-sectional studies and highlighted the potential adverse effect of weight change on depression, particularly weight loss, no considerations of the weight change over the course of several years in different studies were made.

In addition, the other meta-analysis performed for the 15 studies by Luppino FS, de Wit LM, Bouvy PF, et al. [8] found that obesity increased the risk of depression among Americans and Europeans, yet the meta-analysis only included Western participants, and no considerations of other ethnicities, such as Asians. Moreover, there was no analysis focusing on underweight participants. It was uncertain whether underweight affects depression in nationwide cohort participants or whether obesity was associated with depression. Thus, further studies among the middle-aged and older adults in Asian nations are needed to identify whether the relationship between weight change and incidence of depression in Asian participants differs from that observed in Western participants.

To address these gaps, we used four years of longitudinal data from the nationally representative sample of community-dwelling Chinese participants aged45 years and explored to examine the relationship between weight changes, including loss and gain, and incidence of depressive symptom during short–term (two years), long-term (four years), and subsequent term (two years) internals. Furthermore, our study explores the stability of the association between weight changes and depressive symptom by sex on Asians.

## Materials and methods

### Study participants

**Fig. 1:**
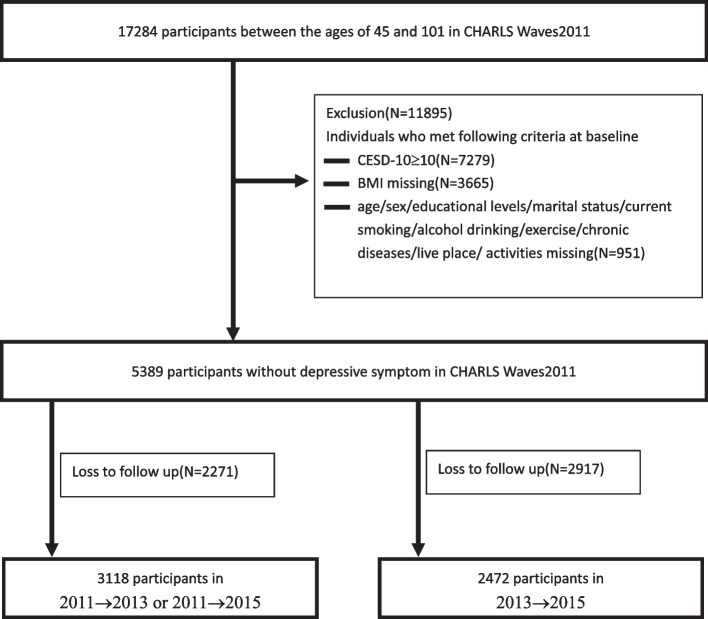
Flow chart of the study participants

We obtained the data from The China Health and Retirement Longitudinal Study (CHARLS). The CHARLS began in 2011 with a cohort of 17,284 participants ≥ 45 years (Wave1). Subsequently, data collection was conducted in 2013(Waves2), 2015(Waves3), and 2018(Wave4). CHARLS was a nationally representative longitudinal survey of the mid-aged and older adults in China along with their spouses. The respondents will be followed every two years using a face-to-face, computer-aided personal interview (CAPI) and structured questionnaire. The current study used data from participants who participated in Wave1, Waves2, and Waves3. We excluded individual who met any of the following criteria at baseline (1) CESD-10 scores 10, (2) no BMI data, (3) no age/sex/educational levels/marital status/smoking status/alcohol consumption/exercise/diseases/live place/activities data. In addition, we excluded participants with no follow-up data. The numbers of individuals who completed both the baseline and follow-up surveys were 3118 for the short-term (2011–2013) and the long-term (2011–2015). Additionally, to associate weight change with subsequent depressive symptoms [associate the weight changes between 2011 and 2013 (exposure) with development of depression between 2013 and 2015(outcome)], we also included 2472 participants without depressive symptom in 2013 and observed the incidence of depressive symptom in 2015 (subsequent term 2013–2015). Figure 1 shows a flow diagram of the study individuals, follow-up, and lost to follow-up.

### Definition of depressive symptom

Based on the Chinese version of the Center for Epidemiologic Studies-Depression scale (CES-D), we used CES-D to define depressive symptom. It consists of 10 items, the total score ranges from 0 to 30, with a higher total score indicating more severe depressive symptoms. We used harmonized criteria cutoff values (total scores ≥ 10) to define depressive symptom. In our study, the Cronbach alpha coefficient was 0.86 and the construct validity was 0.62 [9, 10]. The Chinese version of the CES-D has shown good reliability and validity in the middle-aged and older adults.

### Weight changes

Height was measured by the vertical height meter, and weight was measured with a digital weight scale. Body mass index (BMI) was calculated as weight in kilograms divided by height in meters squared in our study. We used three weight changes indicators according to the interval over which the changes were assessed: short-term (two years from 2011 to 2013), long-term (four years from 2011 to 2015) and subsequent term (two years from 2013 to 2015). The percentages of weight changes between the baseline and the follow-up surveys, based on the baseline body mass index, was calculated. We classified the percentages of weight changes into five categories [4]: loss > -3%, stable-3–3%, gain3-6%, gain6-9%, and gain > 9%.

### Covariates

Covariates included age, sex, education, marital status, current residence, smoking status, alcohol consumption, exercise, diseases, activities at baseline, and entry wave (Wave 1, 2, 3) were incorporated as covariates in our study. Age was categorized into four groups: 45–54, 55–64, 65–74, and ≥ 75 years old. Sex was classified into two categories: male and female. Education was defined as illiterate, or less than elementary school, or high school, or above vocational school. Marital status was defined as the married or the single (divorced, and never married, widowed, or separated). Current residence was categorized into the rural and urban. Smoking status was defined as never smoker or former-smoker or current smoker. Alcohol consumption was categorized into the never drinker, less than once a month drinker, and more than once a month drinker. Exercise was categorized three groups: no exercise, less than regular exercises, and regular exercises. Taking activities including (1) communicating with friends or providing help to neighbors, friends, or family, or (2) doing a sport, social, or other kind of club or playing Ma-jong, or (3) playing played cards, chess, or going to community club, or (4) taking part in a community-related organization, or (5) doing voluntary or charity work, or (6)caring for a sick or disabled adult, or (7) attending an educational or training course, or (8) stock investment, or (9)using the Internet were dichotomized as ever (at least once a month) or never. Diseases included (1) cancer or malignant tumor, (2) dyslipidemia, (3) hypertension, (4) chronic lung diseases, (5) diabetes or high blood sugar, (6) liver disease, (7) kidney disease, (8) asthma were reported by the respondents, (9) arthritis or rheumatism, (10) stomach or other digestive disease, (11) heart attack, coronary heart disease, angina, congestive heart failure, or other heart problems, (12) emotional, nervous, or psychiatric problems, (13) memory-related disease, (14) stroke. According to our previous standard [5], a continuous variable was used to reflect presence of chronic health conditions which ranged from 0 to 14. Numbers of the diseases condition were classified into three categories: 0, 1–2, and 3–14. The categories have been widely used in our studies [11–16].

### Statistical analysis

Statistical analyses were performed using IBM SPSS version21.0 (IBM Corp., Armonk, NY). Categorical variables were expressed as frequencies and percentages and compared using the ^2^ test. Multivariable-adjusted cox regression was conducted using the hazard ratio (HR), and 95% confidence interval (CI) for the association of body mass index (BMI) changes categories with the incident depressive symptom.* P* < 0.05 was considered statistically significant.

## Results

3118 participants who completed the questionnaires effectively were included in the short-term (2011–2013) and the long-term (2011–2015). 2472 participants without depressive symptom were included in 2013(2013–2015). The characteristics of the study participants were shown in Table 1. At baseline, 54.49% of the participants were males in the 2011–2013 and 2011–2015, and 56.59% was males in the 2013–2015. The mean age was 59.15 years (standard deviation [*SD*] = 8.50) in the 2011–2013 and 2011–2015, and was 60.12 years (standard deviation [*SD*] = 8.66) in the 2013–2015. The mean BMI was 23.80 kg/m^2^ (standard deviation [*SD*] = 3.85) in the 2011–2013 and 2011–2015, and 24.15 kg/m^2^ (standard deviation [*SD*] = 3.60) in the 2013–2015.

**Table 1:** Baseline characteristics in CHARLS Waves

Variables	2011–2013/2015 (*N* = 3118,%)	2013–2015 (*N* = 2472, %)
Age(years)		
45–54	36.91	36.08
55–64	40.41	40.86
65–74	19.08	19.30
≥ 75	3.59	3.76
Sex		
Male	54.46	56.47
Female	45.54	43.53
Education		
Illiterate	20.24	19.38
Less than elementary school	66.84	67.15
High school	8.92	9.18
Above vocational school	4.01	4.29
Marital status		
Single	8.05	7.85
Married	91.95	92.15
Current residence		
Rural	8.11	8.86
Urban	91.89	91.14
Smoking status		
No	57.41	56.39
Former smoke	8.60	8.98
Current smoke	34.00	34.63
Alcohol consumption		
No	63.53	62.50
Less than once a month	8.47	8.41
More than once a month	28.00	29.09
Having regular exercises		
No exercise	60.04	60.03
Less than regular exercises	19.88	19.74
Regular l exercises	20.08	20.23
Taking activities		
No	45.73	44.66
Yes	54.27	55.34
Chronic disease(counts)		
0	40.89	41.83
1–2	47.02	46.72
3–14	12.09	11.45

Baseline characteristics classified according to subsequent onset of depressive symptoms were illustrated in Table 2. Participants who developed depressive symptoms were more likely to be female, to have lower educational levels, and be never drinking in the three different sets. They tended to take activities, live in rural, and have 0–2 diseases in the 2011–2013 and 2011–2015. Additionally, they also tended to be never smoking in the 2011–2015 and 2013–2015.

**Table 2:** Baseline characteristics classified according to subsequent onset of depressive symptoms

Variables	2011–2013 incidence depressive symptom (*N* = 3118,%)		P1	2011–2015 incidence depressive symptom (*N* = 3118,%)		P2	2013–2015 incidence depressive symptom (*N* = 2472,%)		*P*_*3*_
	No	Yes		No	Yes		No	Yes	
Age(years)			0.254			0.102			0.653
45–54	892(77.50)	259(22.50)		823(71.5)	328(28.5)		681(76.35)	211(23.65)	
55–64	1010(80.16)	250(19.84)		911(72.3)	349(27.7)		772(76.44)	238(23.56)	
65–74	477(80.17)	118(19.83)		457(76.81)	138(23.19)		377(79.04)	100(20.96)	
≥ 75	93(83.04)	19(16.96)		84(75.00)	28(25.00)		73(78.49)	20(21.51)	
Sex			**< 0.001**			**< 0.001**			**< 0.001**
Male	1399(82.34)	300(17.66)		1327(78.10)	372(21.90)		1129(80.70)	270(19.3)	
Female	1073(75.62)	346(24.38)		948(66.81)	471(33.19)		774(72.13)	299(27.87)	
Education			**0.050**			**< 0.001**			**0.001**
Illiterate	479(75.91)	152(24.09)		424(67.19)	207(32.81)		346(72.23)	133(27.77)	
Less than elementary school	1660(79.65)	424(20.35)		1536(73.70)	548(26.30)		1287(77.53)	373(22.47)	
High school	227(81.65)	51(18.35)		212(76.26)	66(23.74)		175(77.09)	52(22.91)	
Above vocational school	106(84.8)	19(15.20)		103(82.40)	22(17.60)		95(89.62)	11(10.38)	
Marital status			0.417			0.866			0.516
Single	2278(79.46)	589(20.54)		2093(73.00)	774(27.00)		1750(76.82)	528(23.18)	
Married	194(77.29)	57(22.71)		182(72.51)	69(27.49)		153(78.87)	41(21.13)	
Current residence			**0.003**			**0.033**			0.055
Rural	2253(78.64)	612(21.36)		2076(72.46)	789(27.54)		1723(76.48)	530(23.52)	
Urban	219(86.56)	34(13.44)		199(78.66)	54(21.34)		180(82.19)	39(17.81)	
Smoking status			0.060			**< 0.001**			**0.008**
No	1394(77.88)	396(22.12)		1251(69.89)	539(30.11)		1041(74.68)	353(25.32)	
Former smoke	222(82.84)	46(17.16)		205(76.49)	63(23.51)		177(79.73)	45(20.27)	
Current smoke	856(80.75)	204(19.25)		819(77.26)	241(22.74)		685(80.02)	171(19.98)	
Alcohol consumption			**0.029**			**< 0.001**			**< 0.001**
No	1545(77.99)	436(22.01)		1388(70.07)	593(29.93)		1156(74.82)	389(25.18)	
Less than once a month	208(78.79)	56(21.21)		189(71.59)	75(28.41)		156(75.00)	52(25.00)	
More than once a month	719(82.36)	154(17.64)		698(79.95)	175(20.05)		591(82.2)	128(17.8)	
Having regular exercises			0.880			0.812			0.885
No exercise	1484(79.27)	388(20.73)		1371(73.24)	501(26.76)		1147(77.29)	337(22.71)	
Less than exercises	488(78.71)	132(21.29)		446(71.94)	174(28.06)		372(76.23)	116(23.77)	
Regular exercises	500(79.87)	126(20.13)		458(73.16)	168(26.84)		384(76.80)	116(23.20)	
Taking activities			**0.019**			**0.021**			0.127
No	1104(77.42)	322(22.58)		1012(70.97)	414(29.03)		834(75.54)	270(24.46)	
Yes	1368(80.85)	324(19.15)		1263(74.65)	429(25.35)		1069(78.14)	299(21.86)	
Chronic disease(counts)			**0.032**			**0.003**			0.226
0	1034(81.1)	241(18.90)		961(75.37)	314(24.63)		811(78.43)	223(21.57)	
1–2	1155(78.79)	311(21.21)		1063(72.51)	403(27.49)		883(76.45)	272(23.55)	
3–14	283(75.07)	94(24.93)		251(66.58)	126(33.42)		209(73.85)	74(26.15)	
BMI changes			0.194			0.468			0.459
> -3%	436(75.69)	140(24.31)		1027(74.58)	350(25.42)		873(78.72)	236(21.28)	
-3–3%	1109(80.54)	268(19.46)		412(71.53)	164(28.47)		327(75.00)	109(25.00)	
3–6%	462(79.25)	121(20.75)		418(71.7)	165(28.3)		350(75.76)	112(24.24)	
6–9%	223(79.36)	58(20.64)		199(70.82)	82(29.18)		168(75.34)	55(24.66)	
> 9%	242(80.4)	59(19.6)		219(72.76)	82(27.24)		185(76.45)	57(23.55)	

The association between weight changes and incidences of depressive symptoms by baseline BMI were shown in Table 3. Firstly, depressive symptoms risk was increased for BMI loss > 3% during the 2011–2013 [1.329(1.053,1.676)] among the total people. After adjusting for age, educational levels, marital status, smoking status, alcohol consumption, exercise, diseases, live place, and activities, similar results were found in the total population [1.305(1.031,1.651)]. Secondly, depressive symptoms risk was increased for BMI lost > 3% during 2013–2015[1.654(1.153,2.373)] among males, and its effect was stronger for the 2011–2013. After adjusting for age, sex, educational levels, marital status, smoking status, alcohol consumption, exercise, diseases, live place, and activities, depressive symptoms risk was also increased for BMI lost > 3% during the 2013–2015[1.643(1.140,2.368)], and its effect was also stronger than the 2011–2013. Lastly, there was no significant association between weight gain and incident depressive symptom, and no significant interaction effect in terms of the sex*weight changes.

**Table 3:** Hazard rations(95%CI) for new-onset depressive symptom according to changes in BMI

Sex	BMI changes	2011–2013 Incidence rate (%)	HR (95%CI)			2011–2015 Incidence rate (%)	HR (95%CI)			2013–2015 Incidence rate (%)	HR (95%CI)		
			**Unadjusted**	**Age-adjusted**	^**b**^**M-adjusted**		**Unadjusted**	**Age-adjusted**	^**b**^**M-adjusted**		**Unadjusted**	**Age-adjusted**	^**b**^**M-adjusted**
**Female**	> -3%	28.11	1.384(0.999,1.917)	1.404(1.013,1.946)	1.389(0.997,1.935)	36.57	1.299(0.984,1.714)	1.318(0.998,1.741)	1.302(0.984,1.724)	25.25	0.867(0.594,1.265)	0.876(0.599,1.279)	0.877(0.599,1.285)
	-3–3%	22.03	1.000	1.000	1.000	30.74	1.000	1.000	1.000	28.04	1.000	1.000	1.000
	3–6%	25.84	1.233(0.880,1.728)	1.230(0.878,1.723)	1.258(0.895,1.768)	32.65	1.092(0.772,1.545)	1.079(0.762,1.528)	1.080(0.761,1.532)	30.81	1.143(0.794,1.646)	1.140(0.791,1.642)	1.146(0.794,1.654)
	6–9%	27.08	1.315(0.867,1.994)	1.316(0.867,1.997)	1.343(0.881,2.047)	35.77	1.254(0.848,1.855)	1.245(0.841,1.843)	1.244(0.839,1.845)	28.57	1.027(0.641,1.644)	1.031(0.644,1.651)	1.028(0.640,1.651)
	> 9%	21.23	0.954(0.613,1.485)	0.962(0.618,1.498)	0.953(0.608,1.493)	32.88	1.103(0.749,1.626)	1.105(0.749,1.629)	1.109(0.750,1.641)	26.09	0.906(0.570,1.441)	0.914(0.575,1.455)	0.911(0.571,1.452)
**Male**			**Unadjusted**	**Age-adjusted**	^**b**^**M-adjusted**		**Unadjusted**	**Age-adjusted**	^**b**^**M-adjusted**		**Unadjusted**	**Age-adjusted**	^**b**^**M-adjusted**
	> -3%	20.68	1.221(0.873,1.709)	1.220(0.872,1.706)	1.263(0.902,1.767)	22.74	1.035(0.775,1.381)	1.033(0.774,1.379)	1.041(0.777,1.394)	24.79	**1.654(1.153,2.373)** ^*^	**1.656(1.154,2.376)** ^*^	**1.643(1.140,2.368)** ^*^
	-3–3%	17.59	1.000	1.000	1.000	22.15	1.000	1.000	1.000	16.62	1.000	1.000	1.000
	3–6%	16.46	0.923(0.651,1.308)	0.921(0.650,1.306)	0.933(0.656,1.326)	17.79	0.760(0.528,1.096)	0.760(0.527,1.096)	0.745(0.515,1.077)	19.32	1.202(0.831,1.737)	1.203(0.832,1.739)	1.231(0.848,1.787)
	6–9%	13.87	0.754(0.450,1.266)	0.752(0.448,1.263)	0.748(0.444,1.260)	21.19	0.945(0.617,1.449)	0.947(0.618,1.451)	0.961(0.624,1.478)	21.19	1.349(0.829,2.196)	1.353(0.831,2.203)	1.327(0.811,2.172)
	> 9%	18.06	1.033(0.660,1.617)	1.033(0.660,1.617)	1.028(0.658,1.607)	26.24	1.251(0.827,1.890)	1.250(0.827,1.889)	1.176(0.775,1.785)	21.26	1.355(0.845,2.173)	1.353(0.844,2.170)	1.333(0.825,2.152)
**Total**			**Unadjusted**	**Age-adjusted**	^**c**^**M-adjusted**		**Unadjusted**	**Age-adjusted**	^**c**^**M-adjusted**		**Unadjusted**	**Age-adjusted**	cM-adjusted
	> -3%	24.31	**1.329(1.053,1.676)** ^*^	**1.331(1.055,1.680)** ^*^	**1.305(1.031,1.651)** ^*^	29.22	1.181(0.969,1.439)	1.192(0.978,1.454)	1.171(0.957,1.433)	25.00	1.233(0.951,1.599)	1.236(0.953,1.603)	1.210(0.929,1.574)
	-3–3%	19.46	1.000	1.000	1.000	25.91	1.000	1.000	1.000	21.28	1.000	1.000	1.000
	3–6%	20.75	1.084(0.852,1.378)	1.077(0.846,1.370)	1.083(0.849,1.381)	24.28	0.917(0.715,1.175)	0.915(0.714,1.173)	0.908(0.706,1.168)	24.24	1.184(0.916,1.530)	1.180(0.913,1.526)	1.186(0.914,1.537)
	6–9%	20.64	1.076(0.783,1.480)	1.069(0.777,1.469)	1.039(0.754,1.433)	28.13	1.119(0.842,1.488)	1.109(0.834,1.475)	1.092(0.818,1.458)	24.66	1.211(0.865,1.696)	1.209(0.863,1.693)	1.167(0.830,1.640)
	> 9%	19.60	1.009(0.737,1.381)	1.011(0.738,1.384)	0.968(0.705,1.329)	29.62	1.203(0.908,1.595)	1.204(0.908,1.596)	1.133(0.851,1.508)	23.55	1.140(0.819,1.585)	1.145(0.823,1.592)	1.083(0.775,1.512)
**Interaction**			**Unadjusted**	**Age-adjusted**	^**b**^**M-adjusted**		**Unadjusted**	**Age-adjusted**	^**b**^**M-adjusted**		**Unadjusted**	**Age-adjusted**	^**b**^**M-adjusted**
	Sex^*^weight changes		1.033(0.995,1.073)	1.030(0.992,1.070)	1.006(0.964,1.049)		1.064(1.028,1.101)	1.060(1.024,1.097)	1.019(0.981,1.058)		1.067(1.025,1.111)	1.066(1.023,1.110)	1.029(0.983,1.077)

^b^M include age, educational levels, marital status, smoking status, alcohol consumption, exercise, diseases, live place, activities

^c^M include age, sex, educational levels, marital status, smoking status, alcohol consumption, exercise, diseases, live place, activities

^*^P < 0.05

## Discussion

Previous studies have reported differences in the relationship between weight change and the incidence of depressive symptom. Further, the results in the association among the mid-aged and older adults in China have been sparse. Our study used a nationally representative sample of community-dwelling mid and older adults followed for up to four years and explored the relationship between weight change and incidence of depressive symptom with an improved methodology.

The analyses were limited to participants who attended both the 2-year and the 4-year follow-up session, and 3118 individuals for the short-term (2011–2013) and the long-term (2011–2015) were included. We found that weight loss of > -3% during short-term was related to an increased risk of incidence of depressive symptom among total participants. However, this was not completely observed in the subgroup analysis by sex. The reason may be that the statistical power was lower in sex-stratified analyses. Additionally, we have failed to find the associations between weight changes and the incidence of depressive symptom in others subgroups, especially the weight gain groups. Furthermore, in order to associate weight change with subsequent depressive symptoms [associate the weight changes between 2011 and 2013 (exposure) with development of depression between 2013 and 2015(outcome)], we also included 2472 participants without depressive symptom in 2013 and observed the incidence of depressive symptom in 2015 (subsequent term 2013–2015). It was suggested that depressive symptoms risk was increased for BMI loss > 3% during the 2013–2015 among males. However, it was found that weight gain was not associated with the incident depressive symptom. Several clinical trials [17–19] found that unhealthy weight control targeting weight loss increased depression incidence in participants. These associations can be explained, in part, by mental stress triggered by unsuccessful weight control. As weight is considered as an individual and controllable responsibility, difficulty weight loss may be misattributed to personal failure rather than the behavioral weight-loss interventions. Societal pressures with respect to slender body can also moderate associations between weight loss and depressive symptom [20, 21]. Moreover, evidences [22, 23] have supported relative associations of overvaluation, body dissatisfaction, disordered, eating and preoccupation with psychological distress and eating disorder behaviors. In the meta-analysis [7], it has been reported that underweight at baseline increased the risk of incidence depression [1.16(1.08–1.24)]. In contrast to a large number of studies [24–29] that focused on the mechanisms that underlie the relationship between body mass index and depression, there are relatively few studies that investigated the association between weight change and the incidence of depression. Previous studies [30–32] reported that people who were may have low self-esteem. Low self-esteem may portray them as helpless and incapable to cope with the world, and lead to depression [33–37]. In modern society, being underweight may express a poor body image and increase the incidence of depression in males. However, underweight people also increased the incidence of depressive symptom in our study (the 2011–2013 among the total and 2013–2015 among males), and there may be due to leptin in the association during those terms. Leptin [38] can influence the relationship between body weight and depressive symptom. Leptin levels are decreased in participants with low body mass index. In addition, leptin is regarded as an effective antidepressant, and there is a significant correlation between low leptin levels and depressive symptoms [39]. Though several studies [40–44] have found that obesity increased the risk of depression and the result is consistent with meta-analysis [7, 8]. However, Goes VF, Wazlawik E, D'Orsi E, et al. [45] conducted a population-based cohort study including 1,702 older adults in Southern Brazil evaluated between 2009/10 and 2013/14 and found that older adults with obesity had a higher prevalence odds ratio of being depressed than individuals with normal weight while overweight individuals had no significant association with incident depression. The different results reflect that the association between weight and the incidence of depressive symptom may be influenced by the cultural background and related social pressures. Partial results in our study were not in accord with the results of other studies, but those provided insight into associations between weight changes and the incidence of depressive symptom during different terms in the middle-aged and older adults.

## Strengths and limitations of the study

This study has several strengths. First, the weight change was measured by BMI, and the BMI was calculated by the body weight and height which was measured by vertical height meter and digital weight scale when it was objectively measured. Second, the study was based on a nationwide cohort survey, which included participants aged 45 years. Third, it compared the effect of weight changes across two different intervals on the depressive symptom. Previous studies used only set single interval to identify the relationship between weight changes and depressive symptom. It helped us to understand the short- and long-term effects of weight change on the incidence of depressive symptom. At last, we conducted the analysis according to sex, which allowed us to identify sex-specific patterns of relationship between weight change and depressive symptom, and we separately assessed depressive symptom which was related with the loss (> -3%) and gain (3–6%, 6–9%, > 9%) groups. This analysis based on different weight changes provided evidence that the association between weight change and depressive symptom may depend on the sex- and term- patterns in Asia.

Several limitations in our study should be noted. Firstly, the depressive symptom was self-reported in the three waves, when it was subjectively measured. This may have a reporting bias. It is known that people tend to underreport their mental illness in the research. Secondly, we did not consider body composition which was associated with depression [22–24] in several studies. More research should investigate the relationship using indicators of body composition to more fully understand the potential mechanisms linking to weight changes over different time ranges to the incidence of depressive symptom. Thirdly, statistical power was lower in sex-stratified analyses. Fourthly, it was aimed to examine the relationship between weight changes and incidence of depressive symptoms. This will help us to explore the relationship between the depressive symptom and incidence of the weight changes in the next step. Finally, although we have fully adjusted the potential confounders, there was still a chance of residual confounders in the study.

## Conclusions

Our study using a nationally representative sample of community-dwelling older Chinese participants found that weight loss > 3% for intervals was associated with higher depressive symptoms than stable weight during the 2011–2013 [1.329(1.053,1.676)] among the total populations and the 2013–2015[1.643(1.140,2.368)] among the males. Moreover, there was no significant association between weight gain and incident depressive symptom. The result highlights the importance of health promotion interventions to body-weight management aimed at improving the health of older adults, particularly in the total people with short-term weight loss and males with subsequent term weight loss.

## Data Availability

Data can be accessed via http://opendata.pku.edu.cn/dataverse/CHARLS.

## Abbreviations

CHARLS: China Health and Retirement Longitudinal Study
CAPI: Computer-aided personal interview
HRs: Hazard ratios
OR: Odds ratio
BMI: Body mass index
M: Mean
SD: Standard deviation
CIs: Confidence intervals
CESD: Chinese version of the Center for Epidemiologic Studies-Depression scale
NSFC: The National Natural Science Foundation of China
NIA: National Institute on Aging
WB: World Bank
